# From Prebiotics to Mitochondrial Response: A Critical Narrative Review of Microbiota-Host Metabolic Interactions and Their Implications for Child Neurodevelopment

**DOI:** 10.7759/cureus.119347

**Published:** 2026-11-09

**Authors:** Florencia Maria Lucia Sanabria Sanabria

**Affiliations:** 1 Medical Office, Helmar Institute, Mar del Plata, ARG

**Keywords:** autism and gut microbiota, child neurodevelopment, host-microbiota metabolic interactions, mitochondrial bioenergetics, prebiotics

## Abstract

Prebiotics can modify intestinal microbial fermentation and short-chain fatty acid (SCFA) production, but whether these changes improve mitochondrial bioenergetics or neurodevelopmental outcomes in children remains uncertain. This critical narrative review examines the relationships among prebiotic substrates, microbial metabolism, SCFA availability, host metabolic processing, mitochondrial function, and pediatric clinical outcomes. Literature was identified through PubMed/MEDLINE, journal and publisher websites, and citation tracking, with literature identification completed before October 15, 2026. Evidence was organized by intervention or exposure, biological system, and measured endpoint, distinguishing direct microbial metabolite exposure from oral prebiotic supplementation. Experimental studies show that butyrate supports oxidative energy metabolism in colonocytes and that mitochondrial responses to microbial metabolites vary with concentration, tissue, redox state, and individual metabolic capacity. Increased intestinal fermentation does not necessarily establish greater systemic metabolite availability, enhanced mitochondrial ATP synthesis, or improved neuronal function. Pediatric galactooligosaccharide trials have documented microbial changes without directly measuring mitochondrial ATP production or establishing consistent between-group improvements in behavioral or overall gastrointestinal outcomes. Findings from combined probiotic, prebiotic, and synbiotic interventions cannot establish prebiotic-specific efficacy. A hypothesis-generating framework termed fermentation-bioenergetic response uncoupling is proposed, comprising five potential points of divergence: substrate accessibility, microbial transformation, host exposure, metabolic processing, and mitochondrial functional response. Clinical outcomes are assessed separately. Missing downstream measurements indicate an evidence gap rather than demonstrated biological uncoupling. Future pediatric research should integrate chemically defined nutritional interventions, functional microbiome profiling, metabolomics, validated mitochondrial measurements, and prespecified clinically relevant outcomes. Current evidence does not establish prebiotics as treatments for mitochondrial dysfunction or core neurodevelopmental features; potential gastrointestinal benefits require evaluation for specific clinical indications.

## Introduction and background

The intestinal microbiota metabolizes dietary substrates into compounds capable of influencing host physiology. According to the International Scientific Association for Probiotics and Prebiotics (ISAPP), prebiotics are substrates selectively utilized by host microorganisms that confer a health benefit; fermentability alone therefore does not establish prebiotic status [1,2].

Among commonly studied prebiotic substrates, galactooligosaccharides (GOS), fructooligosaccharides (FOS), and inulin-type fructans can influence the generation of short-chain fatty acids (SCFAs), principally acetate, propionate, and butyrate. The resulting metabolite profile depends on substrate characteristics, resident microbial functions, and metabolic cross-feeding among microorganisms [3].

Mitochondrial bioenergetics describes how cells convert available substrates into usable energy, including adenosine triphosphate (ATP) generated through oxidative phosphorylation. SCFAs can act as local energy substrates or metabolic signals. Experimental studies show that butyrate supports oxidative energy metabolism in colonocytes and contributes to interactions between epithelial metabolism and the intestinal environment [4,5]. Findings in intestinal epithelial cells cannot be extrapolated directly to neuronal mitochondria. Human stable-isotope studies also demonstrate that intestinal uptake and first-pass metabolism influence systemic SCFA availability [6].

Mitochondrial responses to microbial metabolites may vary with metabolite concentration, exposure duration, cellular redox state, and host metabolic capacity. Direct propionic acid exposure in human-derived cell lines, together with broader experimental evidence, supports context-dependent mitochondrial effects [7,8]. These findings establish mechanistic plausibility without demonstrating that oral prebiotic supplementation improves mitochondrial ATP synthesis.

This distinction is particularly relevant to pediatric neurodevelopment because neuronal activity, synaptic function, and brain maturation depend on an adequate cellular energy supply. Some children with neurodevelopmental conditions also experience gastrointestinal symptoms, restricted dietary patterns, and nutritional challenges that may influence microbial metabolism. Pediatric studies of galactooligosaccharide (GOS) supplementation in autistic children have reported intestinal microbial changes and exploratory gastrointestinal or behavioral findings, but have not measured mitochondrial ATP synthesis or established consistent improvements in core neurodevelopmental outcomes [9,10].

Diet further complicates interpretation because food preferences can influence microbial composition independently of an autism diagnosis [11]. Inherited metabolic disorders also demonstrate substantial variation in the capacity to process particular metabolites [12]. These considerations support individualized interpretation of microbial and metabolic findings without implying that prebiotics are uniformly harmful or effective across neurodevelopmental populations.

Accordingly, this critical narrative review examines whether changes in prebiotic-mediated fermentation can be linked to measurable mitochondrial outcomes and clinically relevant effects in children. A hypothesis-generating framework termed fermentation-bioenergetic response uncoupling is proposed, organized around five potential points of divergence: substrate accessibility, microbial transformation, host exposure, metabolic processing, and functional expression. The framework distinguishes missing downstream measurements from demonstrated discordance between measured upstream and downstream responses. It is intended to guide research rather than function as a validated clinical or diagnostic tool.

## Review

Methods of literature identification and narrative synthesis

This critical narrative review used targeted literature identification and a structured, mechanism-oriented narrative synthesis. Sources comprised PubMed/MEDLINE, journal and publisher websites, and backward citation tracking from relevant original studies, reviews, and consensus statements. Literature identification was completed before October 15, 2026. The exact calendar day of the final search is unavailable; this temporal boundary is therefore reported without assigning a retrospective search date. The included publications span 1987-2026, covering foundational physiology and contemporary mechanistic and pediatric research.

The search addressed two complementary questions: how prebiotic fermentation and microbial metabolites relate to host mitochondrial function, and whether pediatric prebiotic interventions demonstrate mitochondrial or clinically relevant effects. Search concepts included prebiotics, GOS, FOS, inulin, fermentable fiber, resistant starch, fermentation, acetate, propionate, butyrate, SCFAs, mitochondrial respiration, oxidative phosphorylation, ATP, children, infants, autism, neurodevelopment, gastrointestinal symptoms, and inherited metabolic disease.

Sources were selected for their relevance to a defined step in the substrate-microbial metabolite-host response pathway or to pediatric gastrointestinal and neurodevelopmental outcomes. Mechanistic experiments, human physiological and tracer studies, pediatric randomized trials, relevant reviews, and disease-specific guidance were considered. Adult, animal, and cell studies informed biological plausibility rather than pediatric efficacy. Combined probiotic or synbiotic interventions were analyzed separately from prebiotic-alone interventions.

The narrative synthesis classified evidence by exposure, biological system, design, measured endpoint, and pediatric applicability. Clinical interpretation considered comparators, blinding, sample size, attrition, diet, cointerventions, statistical contrast, and directness of mitochondrial measurement. Between-group treatment comparisons were distinguished from within-group changes and exploratory interactions. Reporting was checked against the Scale for the Assessment of Narrative Review Articles (SANRA) domains for narrative reviews [13]. No systematic-review protocol, duplicate screening, formal study-level risk-of-bias score, certainty grading, or meta-analysis was undertaken. An archived search log was unavailable, limiting reproducibility and precluding claims of exhaustive retrieval.

Evidence categories and limits of inference

The central question is whether a defined prebiotic exposure produces a measurable mitochondrial effect in the host and, separately, a clinically meaningful pediatric benefit. These outcomes occur at different biological levels. A change in bacterial abundance does not demonstrate active fermentation; a fecal SCFA concentration does not measure systemic or cerebral exposure; and a peripheral respiratory assay cannot establish a change in neuronal ATP supply [3,6,14,15]. Table 1 classifies the principal evidence streams according to what each design can and cannot demonstrate.

**Table 1:** Representative evidence classified by exposure, biological system, measured endpoint, and inference limit. Author-developed synthesis. Rows describe different exposure types rather than a graded hierarchy of identical treatments. GOS, galactooligosaccharides; SCFA, short-chain fatty acid; ATP, adenosine triphosphate; MRI, magnetic resonance imaging

Exposure and studies	Design and biological system	Directly measured endpoints	Inference limit
Defined GOS supplementation [9,10]	Pediatric randomized interventions	Microbiota; fecal or urinary metabolites; gastrointestinal and behavioral measures	No mitochondrial respiration, neuronal ATP, or mitochondrial mediation endpoint
Fermentable substrates [16-19]	Human dietary interventions and microbiome modeling	Substrate-specific taxonomic and SCFA-related responses	Does not establish a reproducible benefit for every fiber or a change in host ATP
Directly applied SCFAs [4,7,20,21]	Mouse colonocytes and human-derived cell lines	Oxygen consumption, ATP, or redox-related changes in defined models	Not equivalent to oral prebiotic dosing or cerebral bioenergetics in children
Tracer and compartment studies [6,22,23]	Human physiology, mainly adults	SCFA kinetics, concentration gradients, and exposure	Systemic availability cannot be inferred from fecal concentration alone
Combined microbiota interventions [24-27]	Reviews, synbiotic cohorts, and secondary trial analyses	Microbiota, gastrointestinal, behavioral, or MRI-related outcomes	Multicomponent products do not identify a prebiotic-specific effect
Organic acidemia guidance [12,28]	Clinical consensus for defined enzyme defects	Metabolic vulnerability and disease-specific management	Does not establish harm from fermentable fibers in autism generally

Representative evidence includes defined human fiber interventions and microbiome modeling [16-19]; experimental epithelial and peripheral-cell responses to microbial metabolites [20,21]; human compartment and tracer investigations [22,23]; pediatric evidence syntheses and combined-intervention studies [24-27]; and disease-specific guidance on organic acidemias [28]. These evidence streams address different steps in the proposed pathway and cannot be interpreted as interchangeable measures of prebiotic efficacy.

Substrate specificity, cross-feeding, and the interpretation of fecal metabolites

Prebiotic status requires selective utilization by host microorganisms together with a demonstrated health benefit; fermentability alone is insufficient [1,2,29]. Chemical identity, degree of polymerization, glycosidic linkages, formulation, dose, transit, and resident metabolic capabilities influence which microorganisms can use a substrate. Human studies of chemically different resistant starches and inulin-type fructans have documented distinct community and microbial metabolite profiles, illustrating why effects of one preparation cannot be attributed to all prebiotics [16-19]. Baseline microbiome profiles may modify responses, but functional predictions from community modeling are not equivalent to experimentally measured metabolite flux.

Microbial metabolic cross-feeding adds variability: acetate or lactate generated by one organism may serve as a substrate for another that produces butyrate. Propionate can arise through succinate, acrylate, or propanediol pathways, whose genes are distributed across multiple taxa [3,30-35]. Increased Bifidobacterium abundance therefore does not independently establish increased butyrate production. Metagenomic potential, gene expression, metabolite synthesis, and metabolite utilization require distinct interpretation.

Measured fecal SCFAs reflect the balance among production, microbial reuse, host absorption, metabolism, and intestinal transit. Human studies show substantial concentration gradients between colonic contents and portal, hepatic, and peripheral compartments [6,22,36,37]. Stable-isotope experiments confirm different systemic bioavailabilities for acetate, propionate, and butyrate [6]. A randomized adult inulin trial illustrates that SCFA kinetic changes can be studied without claiming direct effects on cerebral ATP [23]. A higher fecal concentration alone cannot establish greater host exposure or increased mitochondrial substrate delivery.

Additional adult studies help define what microbial changes predict. An acute randomized crossover study did not demonstrate improved endurance performance after inulin supplementation [38]. In a separate exploratory study, inulin-type fructans modified microbial and metabolic measurements in women with obesity [39], while another dietary fiber intervention linked glucose metabolic responses to Prevotella abundance [40]. These interventions involved different substrates, populations, and endpoints; none directly measured mitochondrial ATP synthesis in children or established neurodevelopmental benefit.

Experimental links between microbial metabolites and mitochondrial function

The most direct experimental energetic link concerns intestinal cells. Donohoe and colleagues showed reduced oxidative metabolism and ATP-related activity in colonocytes from germ-free mice, with restoration of aspects of cellular energy metabolism after butyrate exposure [4]. Additional experiments connect butyrate oxidation, epithelial oxygen consumption, hypoxia-inducible signaling, PPAR-γ pathways, and the local ecological environment [5,20,41]. These findings support a local microbiota-colonocyte relationship without demonstrating that a prebiotic increases mitochondrial ATP generation in pediatric neurons.

Metabolite actions outside the colonic epithelium are more heterogeneous. Animal experiments link direct butyrate exposure to energy expenditure and mitochondrial regulatory pathways [42], while human endothelial-cell experiments report mitochondrial effects in a different tissue and inflammatory context [43]. SCFAs can also activate AMP-activated protein kinase and inhibit histone deacetylases in experimental preparations, affecting epithelial barrier proteins, macrophage function, and colonic regulatory T cells [44-47]. These signaling and immune observations should not be equated with an ATP-producing treatment effect.

Direct metabolite studies in human-derived lymphoblastoid cell lines show that mitochondrial responses to propionate and butyrate can depend on concentration, duration, cellular redox conditions, and baseline cellular characteristics [7,21]. A 2026 review of microbial products and mitochondrial function describes both favorable and adverse experimental effects while emphasizing the predominantly preclinical evidence [8]. Increased oxygen consumption may reflect ATP-linked respiration, proton leak, or other processes and is insufficient evidence of improved energetic efficiency. Physiological exposure after oral supplementation must be distinguished from cell-culture concentrations.

SCFAs may influence host physiology through free fatty acid receptor 2 (FFAR2) and G protein-coupled receptor 41 (GPR41) signaling and associated enteroendocrine responses [48,49]. Experimental work and reviews discuss potential neuroepigenetic mechanisms [50], SCFA involvement in gut-brain communication [51], and the broader microbiota-gut-brain axis [52]. In experimental animals, microbiota-associated effects on blood-brain barrier permeability [53] and microglial maturation [54], together with proposed neurodevelopmental windows [55], provide biological plausibility without establishing clinical benefits from pediatric prebiotic supplementation. Neural effects, mitochondrial mechanisms, and clinical treatment effects require separate measurements.

Mitochondrial outcomes and individual metabolic susceptibility

Mitochondrial dysfunction should be defined using an explicit assay and physiological question. Where appropriate, extracellular flux analysis or high-resolution respirometry can quantify basal and maximal oxygen consumption, ATP-linked respiration, proton leak, nonmitochondrial oxygen consumption, and spare respiratory capacity, with standardized cell preparation, substrate conditions, normalization, and replication [14,15]. Direct ATP measurement, membrane potential, redox markers, and mitochondrial biogenesis provide complementary information but are not interchangeable. Improved respiration in cultured peripheral cells cannot be described as increased ATP production in the brain.

Primary mitochondrial disease should not be inferred from nonspecific biomarkers or autism-associated clinical features without appropriate diagnostic evaluation [56]. Propionate metabolism also requires intact host pathways involving propionyl-coenzyme A (CoA), methylmalonyl-CoA, and succinyl-CoA. Experiments in methylmalonyl-CoA mutase deficiency and consensus recommendations for methylmalonic and propionic acidemias illustrate the importance of specific enzyme defects [12,28,57]. These inherited disorders cannot be generalized to autistic children or used to justify empirical broad dietary restriction, antimicrobial treatment, or universal metabolic testing. The relevance of intestinal butyrate transporters, including monocarboxylate transporter 1 (MCT1) and sodium-coupled monocarboxylate transporter 1 (SMCT1), also varies with epithelial and inflammatory status; available work does not establish reliable predictive clinical tests for prebiotic response [58].

Pediatric clinical context gastrointestinal and dietary factors

Constipation, abdominal pain, food selectivity, and nutritional inadequacy are clinically relevant in children with neurodevelopmental conditions. Reviews report a greater burden of gastrointestinal complaints in autism samples, although prevalence estimates are sensitive to participant selection and population differences [59,60]. These conditions warrant independent clinical assessment without establishing a primary mitochondrial defect or demonstrating that a microbiome alteration determines neurodevelopmental characteristics.

Habitual diet is an important potential confounder. Yap and colleagues reported associations among food selectivity, dietary diversity, and microbiome variation; their subsequently published correction should be read alongside the study [11,61]. Dietary restrictions, concomitant medications, stool consistency, gastrointestinal symptoms, and age can influence both microbial profiles and clinical scales. These factors therefore require assessment before microbiota-behavior associations are interpreted as specific treatment responses.

Pediatric randomized studies of prebiotic supplementation alone

Two randomized studies evaluated defined GOS supplementation in autistic children [9,10]. Their populations, outcomes, and statistical analyses differed, and neither directly measured mitochondrial respiration or ATP production. They are considered separately below and summarized in Table 2.

**Table 2:** Pediatric randomized GOS trials and their clinical and mechanistic interpretation. Author-developed synthesis of published trial reports. Follow-up comparisons and comparisons of change use different statistical contrasts. Exploratory interaction P values and within-group trends should not be interpreted as overall between-group efficacy. GOS, galactooligosaccharides; GI, gastrointestinal; 6-GSI, six-item Gastrointestinal Symptom Index; AQ, Autism Spectrum Quotient; ATEC, Autism Treatment Evaluation Checklist; BAMBI, Brief Autism Mealtime Behavior Inventory; SRS-2, Social Responsiveness Scale, Second Edition; QoLA, Quality of Life in Autism Questionnaire

Study and exposure	Allocation and analysis population	Reported comparative outcomes	Critical interpretation
Grimaldi et al., 2018 [9]; 1.8 g/day B-GOS formulation containing 80% GOS; six weeks	41 enrolled; 11 withdrew before randomization; 30 randomized within dietary strata; 26 completed. Existing exclusion and unrestricted diets examined	Taxonomic and metabolomic changes; behavioral signals in diet, treatment, and time interactions. AQ social skills diet × treatment interaction *F*(1, 21) = 4.62, *P* < 0.05; ATEC time × diet × treatment interaction *F*(2, 42) = 3.20, *P* = 0.05. No significant overall gastrointestinal treatment effect	Small exploratory subgroups; diet, attrition, and multiplicity complicate inference; manufacturer involvement. No mitochondrial endpoint
Palmer et al., 2025 [10]; GOS 1.2 g/day in week 1, then 2.4 g/day for five weeks	41 randomized: 22 GOS and 19 placebo; 35 completed; 33 in per-protocol microbiome analyses	At follow-up: SRS-2 *P* = 0.42; BAMBI *P* = 0.17; QoLA A *P* = 0.91 and B *P* = 0.60. Between-group change: total 6-GSI *P* = 0.20; Bifidobacterium *P* = 0.001. Reported GI *d* = 0.47; QoLA B *d* = 0.44	Bifidogenic response without established between-group clinical improvement; small sample and six-week follow-up. No respiration, ATP, or mitochondrial mediation endpoint

Grimaldi et al. enrolled 41 autistic children aged 4-11 years; 11 withdrew during initial procedures, 30 were subsequently randomized within strata defined by existing unrestricted or exclusion diets, and 26 completed the intervention [9]. The six-week intervention used 1.8 g/day of a B-GOS formulation containing 80% GOS versus maltodextrin placebo. Reported taxonomic and fecal or urinary metabolic differences support biological modulation. The behavioral signal arose from diet-treatment and time-related interactions, including a social skills domain, rather than a consistently demonstrated overall treatment effect. Gastrointestinal differences between treatment arms were not significant. Small diet-defined groups, multiple exploratory measurements, attrition, and manufacturer involvement in supply or funding limit clinical confidence. One child withdrew after marked diarrhea and abdominal pain, which is relevant to tolerability [9].

Palmer et al. randomized 41 parent-child dyads, 22 to GOS and 19 to placebo, to GOS or placebo for six weeks in autistic children aged 4-10 years [10]. Dosing began at 1.2 g/day for one week and increased to 2.4 g/day for the remaining five weeks; both groups also received standardized dietetic advice. Thirty-five completed follow-up, and 33 were included in per-protocol microbiome analyses. Between-group comparisons at follow-up did not support differences in social responsiveness (SRS-2, *P* = 0.42), mealtime behavior (BAMBI, *P* = 0.17), or parental quality of life (QoLA Parts A and B, *P* = 0.91 and *P* = 0.60). The between-group difference in change in the total six-item Gastrointestinal Symptom Index was also nonsignificant (*P* = 0.20). Between-group change in relative Bifidobacterium abundance favored GOS (*P* = 0.001). Moderate standardized effect sizes were reported for overall gastrointestinal symptoms (*d* = 0.47) and parental QoL impact (*d *= 0.44), but these do not establish statistically significant clinical treatment effects [10].

In the Palmer study, an observed microbial response did not translate into a clearly demonstrated benefit on the clinical scales during the short trial. This does not demonstrate mitochondrial uncoupling because mitochondrial function was not measured. The 95% intervals reported alongside several scale scores describe group means and cannot substitute for a comparative treatment-effect interval. Nonsignificant results also cannot establish the definitive absence of benefit.

Other pediatric evidence: reviews of synbiotics and nonrandomized studies

Distinguishing a prebiotic intervention from a combined microbiota-targeted intervention is essential. Carnazzo et al. synthesized six randomized trials comprising 1,148 infants aged 0-36 months, of whom 923 contributed at least one gut-brain-axis-related biomarker [24]. Trials included prebiotic, probiotic, and synbiotic interventions. Effects were concentrated in localized microbial or mucosal measurements without robust group-level evidence of improved neurodevelopmental outcomes. Differences in age, intervention mixtures, and the absence of direct mitochondrial endpoints limit inference for GOS treatment in school-aged autistic children.

França Dias et al. identified 15 heterogeneous studies with 1,015 participants in a 2026 systematic review of prebiotic and/or probiotic interventions in autism [25]. Gastrointestinal signals were reported more consistently than behavioral changes, but designs, formulations, concurrent interventions, and outcome instruments varied. Review-level totals may include overlapping primary studies and should not be added to individual trial sample sizes. This synthesis does not establish prebiotic-mediated increases in mitochondrial ATP production.

An uncontrolled longitudinal study of 25 autistic Mexican children reported changes in clinical measures and the microbiome after six months of a multistrain synbiotic [26]. Without a randomized untreated comparator, these changes cannot establish treatment causality or isolate the prebiotic component. In preterm infants, a secondary analysis of a randomized multicomponent supplement containing Bifidobacterium breve, oligosaccharides, and L-glutamine found associations between intestinal microbial metabolic capacity and MRI measures of white matter development [27]. These associations inform future research but neither isolate a prebiotic effect nor demonstrate mitochondrial ATP production or mediation of neurological outcomes. A separate systematic review of propionate, p-cresol, and 4-ethylphenyl sulfate in autism concerns heterogeneous exposures and associations rather than a single causal dietary intervention [62].

Five-gate framework for testing fermentation and bioenergetic responses

The proposed fermentation-bioenergetic response uncoupling framework organizes the exposure-response chain into five assessment points: substrate accessibility, microbial transformation, host exposure, metabolic processing, and mitochondrial functional expression. It is an original synthesis of established processes rather than a newly discovered biochemical pathway, validated syndrome, or clinical decision instrument. Its purpose is to make missing links and alternative explanations explicit, particularly in pediatric intervention research. Here, uncoupling denotes a proposed divergence between fermentation-related exposure and a downstream bioenergetic response; it does not imply proton leak or classical mitochondrial respiratory uncoupling.

An unmeasured mitochondrial endpoint is an evidence gap rather than evidence of biological uncoupling. A study reporting increased Bifidobacterium after GOS without respiratory measurements should be classified as having an unknown downstream mitochondrial response. Evidence of measured discordance would require prespecified paired upstream and mitochondrial endpoints in the same population or relevant model, exposure-appropriate timing, analytical validity, and sufficient precision to distinguish a biologically negligible effect from an inconclusive estimate. Even a nonsignificant mitochondrial result after a significant microbial response does not independently establish mechanistic uncoupling; causal inference requires additional controlled or mediation-based evidence.

Gate 5 concerns a measured mitochondrial bioenergetic response in a defined tissue or cellular system, such as ATP-linked respiration or spare respiratory capacity [14,15]. Gastrointestinal, behavioral, cognitive, and developmental outcomes are assessed separately downstream. Improved respiration does not establish clinical benefit, and symptom improvement does not establish mitochondrial mediation. Clinical outcomes may also arise through immune, endocrine, neural, or other mechanisms [48-52]. Table 3 distinguishes each candidate gate from subsequent clinical validation.

**Table 3:** Five mechanistic gates with paired measurements and interpretation of missing or divergent responses. Author-developed conceptual framework. The final row is an independent clinical assessment rather than a sixth mechanistic gate. Only adequately precise paired measurements can assess biological discordance. The framework is hypothesis-generating and has not been clinically validated.

Gate or assessment stage	Suggested upstream measurement	Corresponding downstream measurement	Interpretation and caveat
1. Substrate accessibility	Defined molecule, dose, adherence, tolerability, and delivery	Evidence of substrate reaching the fermentative compartment	The received dose does not establish microbial utilization.
2. Microbial transformation	Functional pathways, taxa, and substrate depletion	Metabolite production or flux, including cross-feeding	Taxonomic change without flux measurement leaves an incomplete link.
3. Host exposure	Luminal or fecal metabolites and transport context	Systemic kinetics or relevant tissue exposure where feasible	Fecal concentrations alone do not establish systemic availability.
4. Metabolic processing	Cofactors, enzyme phenotype, nutrition, and redox context	Host metabolite processing or context-appropriate intermediates	Similar exposure can produce different host responses; causality requires testing.
5. Mitochondrial functional expression	Appropriate tissue or cell type, standardized exposure, and assay	Prespecified ATP-linked respiration, spare capacity, or direct ATP	Without an assay, the effect is unknown; nonsignificance alone does not establish uncoupling.
Separate downstream clinical outcomes	A priori gastrointestinal symptom or neurodevelopmental outcome	Validated between-group clinical effect and clinically meaningful magnitude	Clinical benefit and mitochondrial response require independent reporting.

Implications for future pediatric studies and limitations

A future pediatric randomized trial should define the exact prebiotic compound and dose, baseline diet, adherence, duration, tolerability, gastrointestinal phenotype, and prespecified clinical question. For gastrointestinal efficacy, a validated symptom score and clinically meaningful between-group difference should be central; for a bioenergetic hypothesis, the principal mechanistic endpoint should directly assess mitochondrial function in an identified tissue or cell type. Microbial taxonomy or fecal SCFAs may serve as candidate mediators but cannot replace a mitochondrial outcome, and peripheral cellular respiration should not be equated with cerebral ATP synthesis.

Where feasible, longitudinal metagenomic or metatranscriptomic data, targeted metabolomics or isotope-based kinetics, and standardized mitochondrial respirometry should be paired in the same participants. Analyses should address baseline diet, medication exposure, gastrointestinal symptoms, developmental age, attrition, multiplicity, and assessment timing. Effect estimates with 95% confidence intervals or appropriate Bayesian credible intervals should be reported for prespecified between-group contrasts. Exploratory subgroups should be labeled, and statistical nonsignificance should not be interpreted as equivalence or a demonstrated absence of effect.

Limitations include heterogeneous substrates, small and short pediatric trials, varied outcomes, mixed interventions, and reliance on adult or experimental evidence. Pediatric prebiotic studies have not measured the complete fermentation-to-mitochondrial pathway. This targeted narrative synthesis lacks an archived search log and formal risk-of-bias assessment. The exact day of the final search could not be specified beyond completion before October 15, 2026. These limitations reduce reproducibility and preclude claims of exhaustive coverage. The framework therefore specifies testable research questions; it does not establish prebiotic efficacy for mitochondrial dysfunction or neurodevelopmental conditions or demonstrate biological uncoupling when downstream measurements are missing.

## Conclusions

The evidence reviewed does not establish that prebiotic-induced microbial changes improve mitochondrial function or neurodevelopmental outcomes in children. Microbial responses, mitochondrial responses, and clinical benefits require separate assessment, and findings from combined interventions cannot establish prebiotic-specific effects.

The proposed five-gate framework identifies testable links between fermentation and mitochondrial bioenergetics. Missing downstream measurements indicate an evidence gap rather than demonstrated uncoupling. Future pediatric trials should pair defined interventions with appropriate mechanistic measurements and clinically meaningful outcomes before therapeutic conclusions are drawn.
